# Prevalence and Determinants of Glaucoma in Citizens of Qatar Aged 40 Years or Older: A Community-Based Survey

**DOI:** 10.4103/0974-9233.80703

**Published:** 2011

**Authors:** Fatma A. Al-Mansouri, Aida Kanaan, Hamad Gamra, Rajiv Khandekar, Shakeel P. Hashim, Omar Al Qahtani, Mohd. Farouk Ahmed

**Affiliations:** Department of Ophthalmology, Hamad Medical Corporation, Doha, Qatar; 1 British Columbia Center for Epidemiologic and International Ophthalmology, University of British Columbia, Vancouver, Canada

**Keywords:** Blindness, Glaucoma, Prevalence Survey, Public Health, VISION 2020

## Abstract

**Background:**

We present the prevalence and determinants of glaucoma among subjects 40 years of age and older in Qatar.

**Materials and Methods:**

This community-based survey was held in 2009 at 49 randomly selected clusters. Demographic details and history of glaucoma was collected by the nurses. Ophthalmologists evaluated the optic disc and retina using a digital camera housed in a mobile van. Visual field was tested with an automated perimeter, the intraocular pressure with an applanation tonometer and the angle of the anterior chamber by gonioscopy. A panel of glaucoma experts diagnosed subjects with glaucoma.

**Results:**

This survey enrolled 3,149 (97.3%) participants. The age- and sex-adjusted prevalence of glaucoma in the population aged 40 years and older was 1.73% (95% confidence intervals [CI] 1.69-1.77). Accordingly, 5,641 individuals in this age group in Qatar would have glaucoma. Chronological age of 60 years and older (Odds ratio [OR] 11.1) and the presence of myopia (OR 1.78) were predictors of glaucoma. Open-angle glaucoma was diagnosed in 44 (65.7%) individuals with glaucoma. In nine (13.4%) and 15 (20.9%) subjects, angle closure glaucoma and other (post-traumatic, pseudoexfoliation) glaucoma were present. Bilateral blindness (vision <3/60) and severe visual impairment (<6/60) were found in four (6%) and three (4.5%) subjects with glaucoma, respectively. Glaucoma was treated in 36 (54%) subjects.

**Conclusions:**

The prevalence of glaucoma among citizens of Qatar aged 40 years and older was 1.71%. Glaucoma was associated with the age of 60 years and older and the presence of myopia.

## INTRODUCTION

Glaucoma is the leading cause of irreversible visual disability.^1^ To eliminate avoidable blindness, the International Agency for the Prevention of Blindness (IAPB) and the World Health Organization (WHO) included glaucoma in the list of priority blinding eye diseases of the VISION 2020 initiative.^2^ Visual disabilities could be prevented in eyes with angle closure glaucoma, and the progress of ocular damage due to open-angle glaucoma can be delayed.^3^,^4^ Therefore, the WHO encouraged member countries to adopt a public health approach to address avoidable blindness due to primary glaucoma. Evidence-based information is required for the proper planning of this initiative.^5^ To our knowledge, data on the magnitude and distribution of glaucoma in the Middle East is limited.

The State of Qatar along with other Gulf Cooperation Council countries endorsed the “VISION 2020” initiative and prioritized the elimination of avoidable blindness.^6^ A national document planning the prevention of blindness was finalized in consultation with the WHO Eastern Mediterranean Regional Office in 2004. The recommendations included evidence-based information on blinding eye diseases.^7^

Community-based data on glaucoma in Qatar is not available. However, ophthalmologists believe that glaucoma comprises a sizable number of visits to the Hamad Medical Corporation (HMC) (Personal communication on 15^th^ July 2009 with Dr F Mansouri, Head of Ophthalmology Department, Hamad Medical Corporation).

Due to a lack of available data, we conducted a survey for blindness (Rapid Assessment of Avoidable Blindness - RAAB), glaucoma and diabetic retinopathy in 2009. In this paper, we present the prevalence and determinants of glaucoma among patients 40 years of age or older in Qatar. Based on the results of the survey, we also propose a public health approach to address glaucoma in Qatar.

## MATERIALS AND METHODS

The research and ethical committee of HMC, Qatar, approved this survey. We obtained the written consent of the health administrators to undertake this survey. The leaders of the civic society gave written permission to undertake this study. As the participants were illiterate, we read out the contents of the consent form, explained the purpose of the study and obtained their verbal consent for eye examination and interviews. The field component of the survey was conducted between January 2009 and April 2009. The glaucoma survey was part of a major survey to determine the magnitude and determinants of visual disabilities. Dineen and colleagues have previously described the methodology behind RAAB.^8^

**Figure 1:**
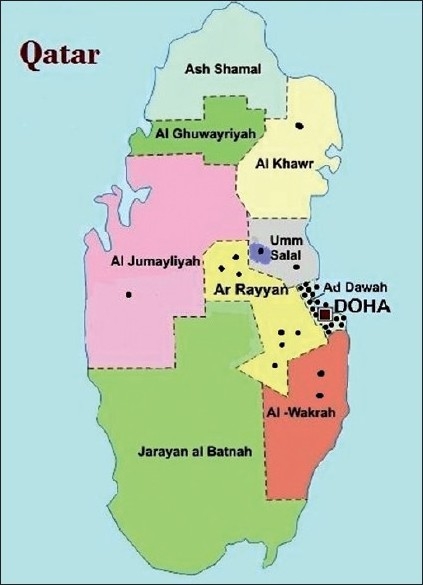
Map of Qatar showing clusters for the glaucoma survey among individuals 40 years of age or older (Glaucoma Survey Qatar 2009)

This was a cross-sectional community-based survey. Citizens of Qatar who were 40 years or older comprised our target population. The residents of randomly selected homes within 49 randomly selected clusters were invited to participate in the survey [Figure 1]. Participants who refused or were absent during the week of the survey in a particular cluster and those severely debilitated and mentally challenged were excluded from the survey.

We assumed that the prevalence of glaucoma in Qatar was 2.1%.^9^,^10^ Our survey population was 326,264 individuals. To achieve a 95% confidence interval (CI) with an acceptable margin of error ranging between 1.4% and 2.8%, we required 1,604 randomly selected subjects. To compensate for the clustering effect, we used a design effect of 2. Hence, the required sample consisted of 3,200 subjects.

We listed the names of the Primary Health Centers with the population of the catchment area in each. We adopted a random selection method based on the Population Proportion to Size (PPS). The names of the clusters were listed and we shortlisted the clusters using the randomization function of Microsoft Excel^®^(Microsoft Corp., Redmond, WA, USA). We planned to include 100 subjects in each cluster.

The survey had three phases. In Phase I, an enumerator visited houses to enroll individuals 40 years of age or older and explained the purpose of the survey. Subjects were requested to visit the ophthalmologist in Phase II of the survey. In a mobile van, the investigators performed vision testing, slit lamp examination, gonioscopy, applanation tonometry, automated visual field testing and digital photography of the disc and the surrounding 30 degrees of the retina.

The enumerators noted general information such as sex, age, name of the cluster and examination status. The examination status was denoted as examined, absent, refused and unable to participate. Ophthalmologists, the nursing staff and volunteers in the selected clusters were our field investigators. They visited a cluster twice a week during the survey period to cover absentees.

An optometrist measured distance visual acuity in natural daylight using the Snellen illiterate “E” chart held at a distance of 6 m from the participant. The participant was asked to wear distance spectacles during this measurement. The best-corrected visual acuity (BCVA) was tested using a pinhole. We used the grades of vision impairment and definitions of eye conditions as recommended by the WHO. The distant vision was graded into ≥6/18, <6/18 to 6/60, <6/60 to 3/60, <3/60, perception of light (PL +) and no PL.

**Figure 2:**
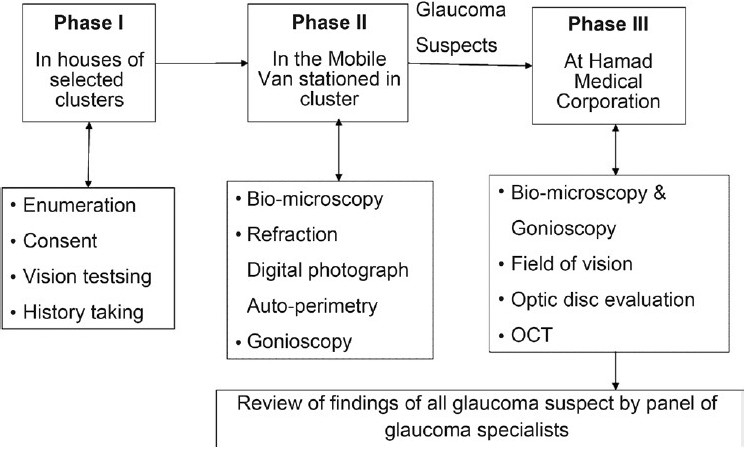
Flow chart showing the steps of assessment of the Qatar glaucoma survey participants (Glaucoma Survey Qatar 2009)

Subjects suspected of having glaucoma in either eye were referred to Phase III of the survey that was held at the Ophthalmology Department of HMC. This visit was conducted at the hospital and all tests of Phase II were repeated, refraction was performed and, if required, Optical Coherent Tomography (OCT) (Carl Zeiss Inc., Jena, Germany) was performed. The tests for glaucoma in different phases are explained in the flow chart [Figure 2].

The ophthalmologist examined all subjects with slit lamp biomicroscopy (Topcon, Japan), digital non-mydriatic fundus images (Carl Zeiss Inc.), indirect ophthalmoscopy (Heine Optotechnik, Herssching, Germany) and gonioscopy (Carl Zeiss Inc.). We graded the angle of the anterior chamber as per the ISGEO classification.^11^ The nursing staff interviewed subjects with a history of glaucoma, glaucoma treatment or glaucoma suspects. Glaucoma was suspected and the subject was referred for further confirmation if either eye had (1) evidence of glaucomatous cup or surrounding retina showed signs of glaucoma or (2) intraocular pressure of ≥22 mmHg measured with applanation tonometry (Carl Zeiss Inc.). In these subjects, if there was no corneal opacity or central lenticular opacity, the visual field was assessed by automated perimetery (Carl Zeiss Inc.). If the results of visual field testing showed glaucomatous field defects that were clinically consistent with the optic cup and surrounding retinal changes, the eye was labeled as glaucomatous.

If the fundus photographs did not match the visual field defect, the subject was considered a glaucoma suspect and was referred to the HMC for further evaluation. OCT was used in four cases of large physiological cups to complement the findings of fundus photography.

The glaucoma survey was part of a major community-based survey that focused on blindness, low vision and diabetic retinopathy in the elderly population in Qatar. The detailed methodology and definitions of various ocular and systemic conditions were defined and printed in the methodology manual prior to the survey. The methodology was tested during a pilot survey held in a cluster outside the study area. The inter-observer agreement among two field ophthalmologists was 0.8 for the interpretation of glaucomatous changes of optic disc and surrounding retina.

A panel of three ophthalmologists (two glaucoma specialists and one general ophthalmologist with experience in a glaucoma clinic) independently reviewed all information of a subject, such as the digital images of both eyes, visual field tests, intraocular pressure, history of treatment and other ocular findings, and labeled the eye and the subject as (1) glaucoma confirmed, (2) not having glaucoma and (3) glaucoma status not conclusive.

The information on blood pressure was collected for the health records of these subjects either from the health center or from the HMC if it was recorded in the previous 3 months. In the remaining cases that visited the HMC, we measured blood pressure with a sphygmomanometer. Hypertension was defined as diastolic blood pressure of more than 90 mmHg provided the subject did not use antihypertensive medication.

To gather high-quality data, a standardization workshop was held prior to the field survey. During the pilot phase inter-observer variation was tested using intraocular pressure and cup to disc ratios as parameters for testing, and the kappa value was 0.8. Instruments were calibrated and maintained to ensure consistency of the measurements. A team of central investigators supervised the field activities.

The data were entered in the computer at the end of the day in the study area. We used EPI6 software (Centers for Disease Control and Prevention, Atlanta, GA, USA) to enter the data. The data were subjected to frequency and consistency checks. It was then transferred to a spreadsheet of Statistical Package for the Social Studies (SPSS Inc., Chicago, IL, USA). We used univariate parametric analysis to calculate the frequencies and the percentage proportions. The magnitude of glaucoma was calculated per person. Based on the crude rates, we estimated individuals with glaucoma in each subgroup, summed them to calculate the age- and sex-adjusted prevalence rates, their 95% CI and the possible number of individuals with glaucoma among the segment of the population that was 40 years and older in Qatar. We used Odd’s ratios (OR) and 95% CI and a two-sided *P*-value to compare the association of different variables to glaucoma.

## RESULT

To represent 326,264 (277,361 males and 48,903 females) citizens who were 40 years and older, we enumerated 3,223 residents of randomly selected 49 clusters. We found 40-100 subjects in clusters.

Of the 3,223 people enumerated, 3,149 (97.7%) persons were examined. Among 74 non-participants, (male [44], female [30], “40-59 years” old [32], “60 years and older” [42], Qatari [47], non-Qatari [27], 30 (0.1%) individuals were absent, 35 (1.1%) refused examination and nine were severely ill or mentally challenged and hence could not be examined. On interview of a relative, we noted that the individuals who were absent or refused to participate had some residual vision and had mobility using their eyesight. In three cases with cataract, we could not get a clear digital image of the optic cup. However, they were screened with a binocular indirect ophthalmoscope. The comparison of population and examined sample by gender and age group is presented in Table 1. The proportion of Qatari and non-Qatari population 40 years or older was not available. However, Qatari nationals comprised of 54.8% of the examined sample. The comparison of the population proportion and proportion of the examined sample in different age groups of males and females suggested that there was a wide variation in representation. Hence, age-sex standardization of the glaucoma prevalence was required for a precise estimate of glaucoma in the community.

**Table 1:** Representation of examined sample to the population (Glaucoma Survey Qatar 2009)

Male					Age group	Female				
Population	% (A)	Examined	% (B)	(A-B)/A*		Population	% (A)	Examined	% (B)	(A-B)/A
18,1215	65.3	377	18.7	0.714	40-49	30,390	62.1	339	29.9	0.519
74,163	26.7	1,048	52.0	-0.945	50-59	10,144	20.7	482	42.5	-1.049
15,051	5.4	383	19.0	-2.503	60-69	4,262	8.7	201	17.7	-1.034
4,688	1.7	171	8.5	-4.021	70-79	2,636	5.4	88	7.8	-0.440
2,244	0.8	36	1.8	-1.208	80+	1,471	3.0	24	2.1	0.296
96,146		2,015			Total	18,513		1,134		

* (A-B)/A is the fraction of the population proportion and examined persons proportion. The value (both negative and positive) suggests gap in representativeness of the study sample in the subgroup

**Figure 3:**
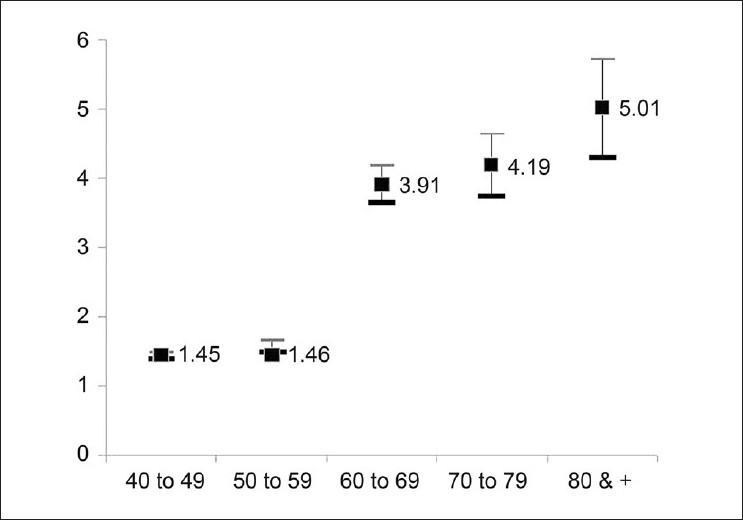
Prevalence of glaucoma by age group in Qatar (Glaucoma Survey Qatar 2009)

In the field, 74 subjects were suspected of having glaucoma and were referred to the HMC for further tests and confirmation. Of these subjects, 67 had glaucoma in at least one eye and the status of the remaining seven subjects was inconclusive, and the subjects were requested to return after 6 months for follow-up. Two of the panel doctors agreed that 65 subjects had glaucoma. In two cases with one eye having doubtful visual field and borderline optic cup changes suggestive of glaucoma, agreement of the third ophthalmologist confirmed the glaucoma status of the subject. The age-sex-adjusted prevalence of glaucoma among citizens 40 years or older was 1.73% (95% CI 1.69-1.77). The prevalence of glaucoma among both sexes by different age groups suggested a rising trend with age. In subjects who were 80 years or older, the sample size was small and the element of chance could not be ruled out [Table 2]. The prevalence of glaucoma by age group is displayed in Figure 3.

**Table 2:** Prevalence of glaucoma among citizens of Qatar who were 40 years of age or older (Glaucoma Survey Qatar 2009)

	Population	Examined	Glaucoma				
			#	Crude rate %	Glaucoma cases*	Adjusted# rate %	95% CI
Qatar	326,264	3,149	67	2.12	5,641	1.73	1.69-1.77
Male	277,361	2,015	50	3.1	5,087	1.83	1.78-1.88
40-49	181,215	377	6	1.6	2,884		
50-59	74,163	1,048	17	1.6	1,203		
60-69	15,051	383	16	4.2	629		
70-79	4,688	171	9	5.3	247		
80+	2,244	36	2	5.6	125		
Female	48,903	1,134	17	2.1	554	1.13	1.04-1.22
40-49	30,390	339	2	0.6	179		
50-59	10,144	482	6	1.2	126		
60-69	4,262	201	6	3.0	127		
70-79	2,636	88	2	2.3	60		
80+	1,471	24	1	4.2	61		
Qatari$	-	1,727	42	2.4	-		1.71-3.16
Other	-	1,422	25	1.8	-		1.08-2.44

* Persons with glaucoma in the ≥40-year-old age group. Citizens of Qatar were estimated based on the adjusted prevalence rate

# Age- and sex-adjusted prevalence rates of glaucoma

$ Prevalence rates for Qatari and other nationals are crude rates

We have compared results of our study to other studies [Table 3].Different definitions and target populations were used to estimate the magnitude of glaucoma in different studies. Our survey had lower rates compared with the community-based prevalence study conducted in Oman.

**Table 3:** Prevalence of glaucoma in different studies (Glaucoma Survey Qatar 2009)

Ref	Country	1^st^ author	Year	Prevalence (%)	Target population	Sample size	Remarks
	Qatar	Present study	2009	1.73	≥40 years old citizen	2,433	Community-based and digital images
8	Bangladesh	Rahman MM	2004	2.1	>35 years	2,347	CD ratio, field testing
9	Spain	Anton A	2004	2.1	40-79 years	569	Disc photograph and automated field test
14	Oman	Khandekar R	2005	4.75	.30 years old Omani	3,324	Community-based and direct ophthalmoscopy
15	Nepal	Sah RP	2007	0.94	>40 years	1,600	Perkins tonometry, Goldman perimetry
16	USA	Lee PP	2003	4.6% - 1991 13.8% - 1999	>60 years	10,476	Surviving Medicare beneficiaries
17	UK	Kroese M (98)	2002	0.98 1.23	40-89		POAG in hospital POAG in community
18	Japan	Iwase A	2004	3.9	>40 years	3,021	Disc photograph and automated field test
19	USA	Coleman A	2001	1.55			Black>whites
20	Thailand	Bourne RR	2003	3.8	>50 years	701	Field test, VCDR
21	South India	Ramakrishnan R	2003	2.6	>40 years	5,150	Field and disc changes
22	South Africa	Rotchford AP	2003	5.3	>40 years	893	
23	Singapore	Shen SY	2008	3.4	40-80	3,280	SLE, Humphry, applanation
24	Myanmar (40)	Casson RJ	2007	4.90	>40 years	2,076	ISGE definition
25	South Brazil	Sakata K	2007	3.4	>40 years	1,636	ISGEO def
26	Rural S India	Vijaya L	2005	1.62	>40 years	3,924	POAG

**Figure 4:**
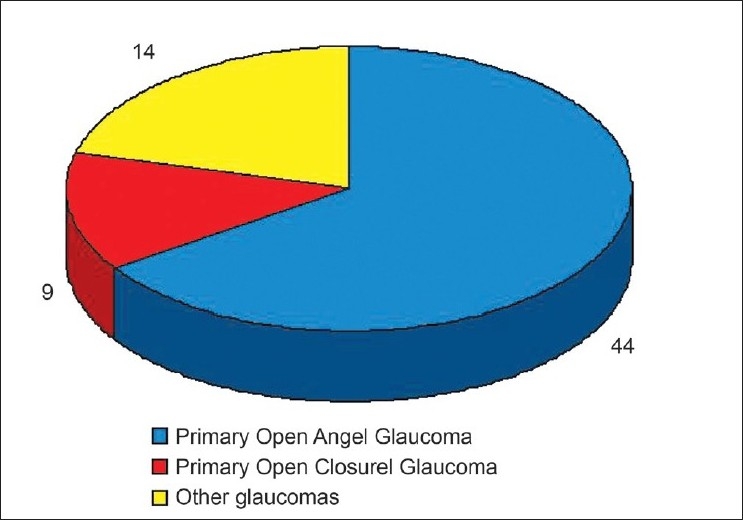
Glaucoma by type in Qatar (Glaucoma Survey Qatar 2009) POAG: Primary open angle glaucoma, PACG: Primary angle closure glaucoma, Other glaucoma include pseudoexfoliation glaucoma and post-traumatic glaucoma

Of the 67 subjects diagnosed with glaucoma, 44 (1.40% [95% CI 0.99-1.81]) had primary open angle glaucoma (POAG), 14 (0.44% [95% CI 0.21-0.68]) subjects had primary angle closure glaucoma (PACG) and nine (0.29% [95% CI 0.21-0.68]) subjects were suffering from other types of glaucoma (post-traumatic, pseudoexfoliation). [Figure 4] Fourteen subjects with PAOG (eight males and seven females, 10 Qatari and five non-Qatari, four subjects from the 50-59 years of age group, seven subjects from the 60-69 years of age group and four subjects from the 70 years and older group) had a mean intraocular pressure of 19.5 mmHg ± 5.9 mmHg.

We analyzed glaucoma and different visual disabilities among ≥50-year-old participants. Data on the vision of subjects aged 40-49 years were not available as the visual acuity for the blindness and low vision study focused on subjects 50 years or older. Of the 38 subjects with vision <3/60 (WHO Blind), 15 (39.5%) subjects had glaucoma as the principal cause. Among 51 legal blind (<6/60), 15 (29.4%) subjects had glaucoma as a principal cause of visual impairment in the better eye. Of the 108 subjects with low vision disability (vision <6/18 best corrected), 11 (10.2%) subjects had glaucoma. There were 107 subjects with unilateral blindness. Ten (9.3%) subjects had glaucoma as the principal cause in the blind eye (vision <3/60).

The risk of glaucoma did differ among males and females (OR = 1.67, 95% CI 1.0-2.9, *P* = 0.03). Subjects in the age group of 60 years and older had a significantly higher risk of glaucoma compared with subjects in the 40-59 years age group (OR = 2.97, 95% CI 1.82-4.82, *P* = 0.00001). The risk of glaucoma among Qatari and non-Qatari citizens was not different (OR = 1.39, 95% CI 0.84-2.29, *P* = 0.09) [Table 4]. Subjects 60 years of age and older with myopia had a positive association with glaucoma.

**Table 4:** Risk factors of glaucoma in Qatar (Glaucoma Survey Qatar 2009)

Variant		Glaucoma	No glaucoma	Odds ratio	95% confidence interval	*P*-value
Gender	Male	50	1,588	1.44	0.83-2.5	0.2
	Female	17	775			
Age group	40-59	31	2,215	11.8	7.2-19.5	<0.001
	60+	36	218			
Myopia	Yes	21	494	1.78	1.06-3.03	0.03
	No	46	1,939			
Hypertension*	Yes	28	8	0.90	0.28-2.90	0.9
	No	39	10			
Glaucoma in family*	Yes	10	1	2.98	0.37-66.7	0.44
	No	57	17			

* The information of hypertension and family history was limited to 110 cases suspected to have glaucoma

Of the 67 subjects with glaucoma, 33 (49.3%) knew that they were suffering from glaucoma, 30 (44.8%) said that they did not have glaucoma while the remaining four subjects were unsure regarding their glaucoma status. Among the subjects with glaucoma, 36 (53.7%) had undergone previous treatment, 27 (77.1%) were using eye drops, two (5.7%) had undergone laser treatment and six (17.2%) subjects had undergone surgery for glaucoma in at least one eye.

## DISCUSSION

Glaucoma is a chronic and age-related non-communicable disease. Hence, its prevalence will rise in countries with an increasing geriatric population. The cases are often symptomless, and glaucoma patients in advanced stages may frequently visit different eye clinics. Therefore, a community-based prevalence rate rather than the rate among patients visiting hospitals is a better indicator of the magnitude of glaucoma.^12^ In undertaking a survey similar to ours, logistics are very complex as ophthalmologists are essential to accurately diagnose glaucoma in the early stages, and they also require modern technology that is difficult to transport to survey sites. We used a mobile van with the required equipment to reach the community and provided comprehensive eye care to the survey participants at their doorstep. This facilitated data collection as we went to the “data site” rather than vice versa.

Reliable population estimates are fundamental to the application surveys such as ours. Based on the 2004 census, the population in Qatar was estimated to be 1.62 million by mid-2008. Based on these projections, the population of Qatar comprised of 25% native Qatari, 35% Arab residents and 40% expatriate laborers.^13^ The laborers undergo health check on recruitment and renewal of their employment contracts. Due to their young age and a periodic health check up system, age-related and chronic non-communicable diseases are less likely in the immigrant labor force. To identify glaucoma in its early stages, a focus on the middle-aged segment of the population is advised. However, the ideal age for initiating glaucoma screening remains contentious. Our study confirmed previous studies that recommend community-based glaucoma surveys targeting the segment of the population aged 40-45 years and older.^14^,^15^

The magnitude of glaucoma also depends on the historic availability of eye services and their utilization. It is therefore crucial to review eye care services in Qatar. Due to the short distances and limited population, ophthalmologists used to provide eye care only at HMC at Doha, Qatar, until the end of the last decade. In 2001, ophthalmic units were established at six primary health centers with large populations in their catchment area. But, facilities in these eye clinics are limited. Hence, most of the patients with the blinding eye diseases are still referred to HMC for final diagnosis and management. Ophthalmic services are also offered at the Armed Forces Hospital and Police Hospital in Doha. In addition, many eye clinics and two hospitals in the private sector now offer eye care in Qatar. Thus, a backlog or lack of service for patients with blinding eye diseases due to a lack of eye care services is less likely.

Based on our outcomes, the prevalence of all types of glaucoma in the segment of the population in Qatar that is 40 years or older was 1.73%. The prevalence of glaucoma worldwide differs significantly, ranging from 0.94% in Nepal to 13.8% in the USA.^1^,^16^–^26^ The higher prevalence in the group from the USA was mainly from one study that focused on a specific high risk population but the average prevalence in other studies were lower than that reported by Lee and colleagues.^16^ Based on our experience, it is important to note the age and race of the target population, the type of equipment and definitions of glaucoma, glaucoma suspect and normal prior to undertaking any comparison. To our knowledge, the data on glaucoma among Arabs, especially at the community level, is rare. A survey in Oman noted a 4.75% prevalence of glaucoma in individuals who were 30 years of age and older. The presence of individuals of African origin in Oman could explain the higher rates compared to the rate in Qatari citizens.^14^ In industrialized countries, surveys employed standard equipment and definitions. However, the glaucoma rate still ranged from 1.23% in a regional UK survey to 3.9% in Japan.^18^,^19^ In Qatar, 5,641 cases of glaucoma were estimated based on a age and sex adjustment in the segment of population that was 40 years or older. The formation of a glaucoma registry and undertaking of opportunistic eye screening for glaucoma and diabetic retinopathy should be considered in Qatar, which is a resource-intense country with a commitment to provide affordable eye care to all and to reach VISION 2020 goals.^6^

There is ample evidence in the literature suggesting a positive association of older age and glaucoma.^27^,^28^Our study also confirmed this association. Surveys and screenings targeting an older age group therefore might take place in the future. However, individuals who are 50 years of age and older comprise only 10% of the total population in developing countries. This should be taken into account before limiting screening of such chronic eye diseases to elderly citizens. Effectively, researchers and clinicians miss the opportunity of early detection of glaucoma by screening individuals aged 40-50 years.

In our study, the age-adjusted prevalence of glaucoma was higher in males compared with females. However, it was not a statistically significant risk factor for glaucoma among participants. Similar observations were made in a meta-analysis covering studies on POAG by Rudnika and colleagues.^29^ In contrast, Cedrone and colleagues found higher rates of glaucoma in females.^30^ The proportion percentage of PACG was smaller than the proportion of POAG in our study in Qatar. The former is known to be more common in females. This could be the reason for higher risk of glaucoma in male compared to female.

The higher prevalence of glaucoma among Qatari compared to non-Qatari nationals in our study is a notable finding. Further investigation is required to verify our results prior to interpreting the association of nationality to glaucoma. Racial differences are observed in glaucoma and familial as well as genetic etiologies have been proposed for different types of glaucoma.^31^,^32^ A high consanguinity rate in the Qatari population may be a factor in this higher prevalence of glaucoma.^33^

POAG comprised 2/3 of the glaucoma patients in Qatar. PACG occurred in <15% of the glaucoma patients. In Oman, the proportion of POAG and PACG was equal.^14^ A study in western Saudi Arabia, a neighboring country, revealed that the proportion of POAG and PACG was 31% and 25%, respectively.^34^ As the outcomes from Saudi Arabia were based on a hospital survey, one should compare the outcomes with the Qatar study cautiously. Caucasians and people of African origin have a higher risk of POAG while Asians and the Chinese population have a higher risk of PACG.^35^–^39^Although race is associated with the type of glaucoma, further studies are needed to conclude about the proportion of the subtypes of glaucoma in the Arab population. Different criteria are used to define glaucoma. Although Qatar is part of the Asian continent, we did not use the South-East Asian classification to define glaucoma. Therefore, a comparison of our study results with other prevalence surveys should be interpreted with caution.

In our study, the association of glaucoma to a positive family history was not statistically significant (*P* > 0.05). This could be due to the inclusion of only glaucoma suspects in the interview to collect family history of glaucoma instead of all participants. The literature has clearly shown a strong familial trait of glaucoma.^40^–^42^

Hypertension and vascular conditions have been documented to have a role in the pathogenesis of POAG.^43^ Unfortunately, our method of collecting information on hypertension was a review of case records and not by physical measurement at the time of assessing glaucoma status. Therefore, this association should be interpreted with caution. Control of hypertension limits visual damage due to glaucoma.^44^ As primary health care is freely accessible to the citizens of Qatar, this strategy could be a part of limiting visual disability due to glaucoma.

Glaucoma in Qatar was significantly associated with myopia. Scientists have shown a strong association of moderate to severe myopia to POAG.^45^,^46^ Axial myopia has also been associated with glaucoma.^24^ Unfortunately, we did not study the relationship between the magnitude of myopia and glaucoma. A study with a larger sample to assess the risk of glaucoma among moderate and severe myopes is recommended. Optometrists are usually involved in the early detection of ocular diseases, refraction and the correction of myopia. They can refer moderate and severe myopes to ophthalmologists for further assessment to rule out glaucoma. Standardized training and providing legal permission for glaucoma screening would be required prior to including an optometrist in a programmed approach for glaucoma detection.

We found that nearly half of the subjects with glaucoma were unaware of having glaucoma - a sight-threatening eye disease. As glaucoma has mild or no symptoms, this is expected. This strongly favors an emphasis on early detection, counseling of glaucoma patients and health education of staff and public education. In comparison, the awareness among patients suffering from glaucoma was poorer in studies conducted in India and Egypt.^15^,^25^,^47^ Free and easily accessible eye care services in Qatar likely explain the better coverage of glaucoma detection.

More than half of the patients had sought treatment for glaucoma. Three-fourth of those treated were using local eye drops at the time of the survey and the other 6% underwent previous surgery. Coverage of glaucoma treatment rather than the rate of glaucoma surgery could be a better indicator to monitor the glaucoma control program.

RAAB in Qatar was conducted along with the glaucoma survey. However, for RAAB, subjects 50 years and older were targeted. Therefore, while studying the role of glaucoma in visual disability, we could focus on glaucoma cases in the segment of the population that was 50 years and older. In this segment of the population, we found that nearly 40% bilateral blindness (WHO definition) was principally due to glaucoma. This was higher than the 10% reported by Cedrone and colleagues and 30% in another study in sub-Saharan Africa.^30^,^48^ In our survey, we added four cases with tubular vision while calculating the rate of blindness. This may explain the higher proportion of blindness due to glaucoma in our survey compared to previous studies.^31^,^48^ Glaucoma caused 30% of legal blindness in Qatar. In contrast, half of the legally blind had glaucoma as the principal cause in Saudi Arabia.^34^ It seems that cases of glaucoma were in advanced stages in Qatar. However, due to a limited number of glaucoma cases in our study, conclusions should be drawn judiciously. Based on these outcomes, we recommend the implementation of programs to detect the early stages of glaucoma cases and ensure a standard management with regular follow-up.

Four cases of glaucoma had tubular visual fields and were detected for the first time during the survey. In a country with easily accessible eye care, this was unexpected. The situation in Qatar seems to be better than that reported in Southern Brazil, where 90% of the glaucoma cases were previously undetected.^25^ This shows the importance of altering health-seeking behavior and increasing awareness among the community about the risk of this asymptomatic sight-threatening eye disease.

The members of the national committee for the Prevention of Blindness of Qatar and research committee of HMC were informed about the study outcomes and policies to strengthen the public health approach for glaucoma. Comprehensive eye examination is recommended to all elderly people that visit eye clinics. In Belgium, universal glaucoma screening was proposed. In view of the large number of cases, researchers in Oman suggested an opportunistic screening instead of universal glaucoma screening, maintenance of a glaucoma registry at eye clinics and maintenance of the follow-up schedule for all glaucoma cases.^14^,^49^

In our survey, we conducted screening of glaucoma and diabetic retinopathy together. This was also suggested by Beyanat and colleagues.^50^ Comprehensive eye examination is recommended for all elderly people.

In this study, we noted a 1.71% prevalence of glaucoma among citizens of Qatar aged 40 years and older. The prevalence of POAG, PACG and other glaucoma was 1.4%, 0.44% and 0.29%, respectively. Glaucoma was associated with the “60 and more” age group and with myopia. Nearly half of the glaucoma patients were unaware that they suffered from glaucoma.
